# Cellular Toxicity of Nanogenomedicine in MCF-7 Cell Line: MTT assay

**DOI:** 10.3791/1191

**Published:** 2009-04-03

**Authors:** Somaieh Ahmadian, Jaleh Barar, Amir Ata Saei, Mohammad Amin Abolghassemi Fakhree, Yadollah Omidi

**Affiliations:** Research Center for Pharmaceutical Nanotechnology, Faculty of Pharmacy, Tabriz University (Medical Sciences); Gifted and Talented Students Office, Educational Development Center, Tabriz University (Medical Sciences); School of Advanced Biomedical Sciences, Tabriz University (Medical Sciences)

## Abstract

Cytotoxicity of the futuristic nanogenomedicine (e.g., short interfering RNA and antisense) may hamper its clinical development. Of these, the gene-based medicine and/or its carrier may elicit cellular toxicity. For assessment of such cytotoxicity, a common methodology is largely dependent upon utilization of the 3-(4, 5-Dimethyl-2-thiazolyl)-2, 5-diphenyl-2H-tetrazolium bromide (MTT) assay which has been widely used as a colorimetric approach based on the activity of mitochondrial dehydrogenase enzymes in cells. In this current investigation, MCF-7 cells were inoculated in 96-well plate and at 50% confluency they were treated with different nanopolyplexes and subjected to MTT assay after 24 hours. Water soluble yellow MTT is metabolized by the metabolically active cells to the water insoluble purple formazan, which is further dissolved in dimethylsulfoxide and Sornson s buffer pH 10.5. The resultant product can be quantified by spectrophotometry using a plate reader at 570 nm.

**Figure:**
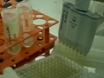


## Protocol

### MCF-7 seeding in 96-well plate:

MCF-7 cells were cultured in 25 t-flask in medium containing Dulbecco’s Modified Eagle’s Medium (DMEM), 10% FBS, 100 U/ml penicillin, and 100 μg/ml streptomycin at 37°C with 5% CO_2_, 95% air and complete humidity. Once reached ~90% confluency, they were detached using 0.05% trypsin/EDTA and counted by means of trypan blue and hemocytometer. These cells were then resuspended at a concentration of 4×10^4^ cells/cm^2^ and added onto 96-well plate (i.e., 250 μl/well) by an 8-channel pipette. For background absorption, some wells were remained cell-free, i.e. as blank control.

### Treating cells with different nanopolyplexes:

At 40-50% confluency (48 hours post seeding), the cultivated cells were treated with nanostructured starburst polyamidoamine dendrimers (i.e., Superfect® and Polyfect®) and a novel test polymer following the transfection instruction provided by supplier.  Cells were also treated with EGFR and scrambled antisense alone, and with the three different nanopolyplexes of these two oligonucleotides and with polymers (n=4). Four wells were remained untreated as control. After 4 hours the treatment media were removed and replenished with fresh media.

### MTT assay for evaluating cell viability:

MTT assay was performed 24 hours after transfection. For this purpose, MTT solution was prepared at 1mg/ml in PBS and was filtered through a 0.2 µm filter. Then, 50 µl of MTT plus 200 µl of DMEM without phenol red were added into each well, except the cell-free blank wells. Cells were incubated for 4 hours at 37°C with 5% CO_2_, 95% air and complete humidity. After 4 hours, the MTT solution was removed and replaced with 200 µl of DMSO and 25µl Sorenson’s glycine buffer (glycine 0.1M, NaCl 0.1M, pH:10.5 with 0.1 NaOH). The plate was further incubated for 5 min at room temperature, and the optical density (OD) of the wells was determined using a plate reader at a test wavelength of 570 nm and a reference wavelength of 630 nm.

## Discussion

The MTT assay is deemed to be a versatile method and accordingly the viability of the cells could be evaluated upon various treatments. The production of resultant formazan appears to be proportional to the level of energy metabolism in the cells. Therefore, it is possible to measure the metabolically activated cells even in the absence of cell proliferation. The amount of formazan produced is proportional to the amount of MTT in the incubation medium. While, the concentration of MTT which is required to achieve maximum amount of formazan produced may change upon utilization of different cell lines. Besides, having used this assay, very small number of living cells could be detected and the incidence of errors would be minimal since there is no need for washing steps. The absorption of formazan varies with cell number as well as pH which could be overcome with addition of buffer at pH 10.5 ^1^. The color of formazan is stable for a few hours at room temperature ^2^. In the case of more than one plate, controls should be included in other plates as well. Nevertheless, this method suffers from some minor disadvantages: a) metabolically inactive cells cannot be discriminated with dead cells ^3^, b) MTT solution should be protected from light even though it could be stored at 4°C for a maximum of one month ^2^, c) it fails to validate drug stability in the medium, and d) cells used for MTT can not be subsequently used for any other assays.

It should be evoked that phenol red absorbs at 570 nm. Further, it has been previously reported that the phenol red possesses estrogen activity which may affect the cell growth pattern within some estrogen responsive cells, ensuing imprecise MTT results.  To avoid such impact, we have utilized DMEM without phenol red ^4^.
